# Differential combinatorial regulatory network analysis related to venous metastasis of hepatocellular carcinoma

**DOI:** 10.1186/1471-2164-13-S8-S14

**Published:** 2012-12-17

**Authors:** Lingyao Zeng, Jian Yu, Tao Huang, Huliang Jia, Qiongzhu Dong, Fei He, Weilan Yuan, Lunxiu Qin, Yixue Li, Lu Xie

**Affiliations:** 1 School of Life Science and Technology, Tongji University, Shanghai 200092, P.R.China; 2 Shanghai Center for Bioinformation Technology, Shanghai 200235, P.R.China; 3 Key Lab of Systems Biology, Shanghai Institutes for Biological Sciences, Chinese Academy of Sciences, Shanghai 200031, P.R.China; 4 Liver Cancer Institute and Zhongshan Hospital, Institutes of Biomedical Science, Fudan University, Shanghai 200032, P.R.China

## Abstract

**Background:**

Hepatocellular carcinoma (HCC) is one of the most fatal cancers in the world, and metastasis is a significant cause to the high mortality in patients with HCC. However, the molecular mechanism behind HCC metastasis is not fully understood. Study of regulatory networks may help investigate HCC metastasis in the way of systems biology profiling.

**Methods:**

By utilizing both sequence information and parallel microRNA(miRNA) and mRNA expression data on the same cohort of HBV related HCC patients without or with venous metastasis, we constructed combinatorial regulatory networks of non-metastatic and metastatic HCC which contain transcription factor(TF) regulation and miRNA regulation. Differential regulation patterns, classifying marker modules, and key regulatory miRNAs were analyzed by comparing non-metastatic and metastatic networks.

**Results:**

Globally TFs accounted for the main part of regulation while miRNAs for the minor part of regulation. However miRNAs displayed a more active role in the metastatic network than in the non-metastatic one. Seventeen differential regulatory modules discriminative of the metastatic status were identified as cumulative-module classifier, which could also distinguish survival time. MiR-16, miR-30a, Let-7e and miR-204 were identified as key miRNA regulators contributed to HCC metastasis.

**Conclusion:**

In this work we demonstrated an integrative approach to conduct differential combinatorial regulatory network analysis in the specific context venous metastasis of HBV-HCC. Our results proposed possible transcriptional regulatory patterns underlying the different metastatic subgroups of HCC. The workflow in this study can be applied in similar context of cancer research and could also be extended to other clinical topics.

## Introduction

Hepatocellular carcinoma (HCC) is one of the most hazardous cancers in the world. Metastasis remains a significant cause to the high mortality in patients with HCC. The molecular mechanism underlying the metastasis of HCC has not been completely unraveled due to the complexity and heterogeneity of this disease.

With the technology advances in genomics and proteomics, many attempts have been made to predict HCC metastasis based on molecular profiling from mRNA or miRNA microarrays and mass spectrometry assays, sampled from tumor or adjacent non-tumor liver tissues [1-3]. These studies were mostly conducted by selecting from a list of genes whose expression level discriminated well between different sample types. However, the signatures or biomarkers from independent studies shared little overlap. Moreover, the signatures or biomarkers brought us insufficient knowledge about mechanism of HCC metastasis, despite the conventional gene set enrichment analysis.

In recent years, systematic approaches have improved the understanding of complex diseases from multiple perspectives. A priori knowledge such as protein interactions, pathways, clinical factors, or other disease-related information from databases, integrated with gene signature analysis have helped marker gene prioritization [4-8]. In addition, gene relationships among different disease statuses were investigated through systematic network analyses [9,10]. The signature/biomarker identification was also aided by network analysis, which brought advantages over the previous gene-list approaches in prediction accuracy. In 2007, Chuang et.al identified markers for breast cancer metastasis not as individual genes but as subnetworks extracted from protein interaction databases [11]. The subnetwork markers were proved to be more reproducible than individual marker genes and achieved higher accuracy in the classification. In 2010, Li et.al identified breast cancer prognostic modules extracted from GO-term-defined gene sets with both high predictability of metastasis and rational biological senses [12].

In 2009 Martinez N et.al pointed out the importance about the genome-scale combinatorial regulatory networks involving microRNAs(miRNAs), transcription factors(TFs), and genes [13]. They mapped the first genome-scale TF-miRNA transcription regulatory network in C. elegans and integrated this network with a computationally predicted miRNA-TF post-transcriptional network [14]. They investigated the topology and properties of the network to understand how TFs and miRNAs interact to regulate gene expression. After that, significant progress has been made in studies using gene regulatory network models that capture physical and regulatory interactions between genes and their regulators [15]. In 2009, we also published a preliminary research on the microRNA-driven regulatory mechanisms through the combinatorial regulatory network analysis [16]. In that work we used miRNA perturbed gene expression datasets and developed general miRNA-centered regulatory cascades in human cell lines. Biological context was not of concern then. In recent years, regulatory network analyses were brought into different biological contexts to further understand mechanism of complex diseases such as prostate cancer [9] and schizophrenia [10].

**Figure 1:**
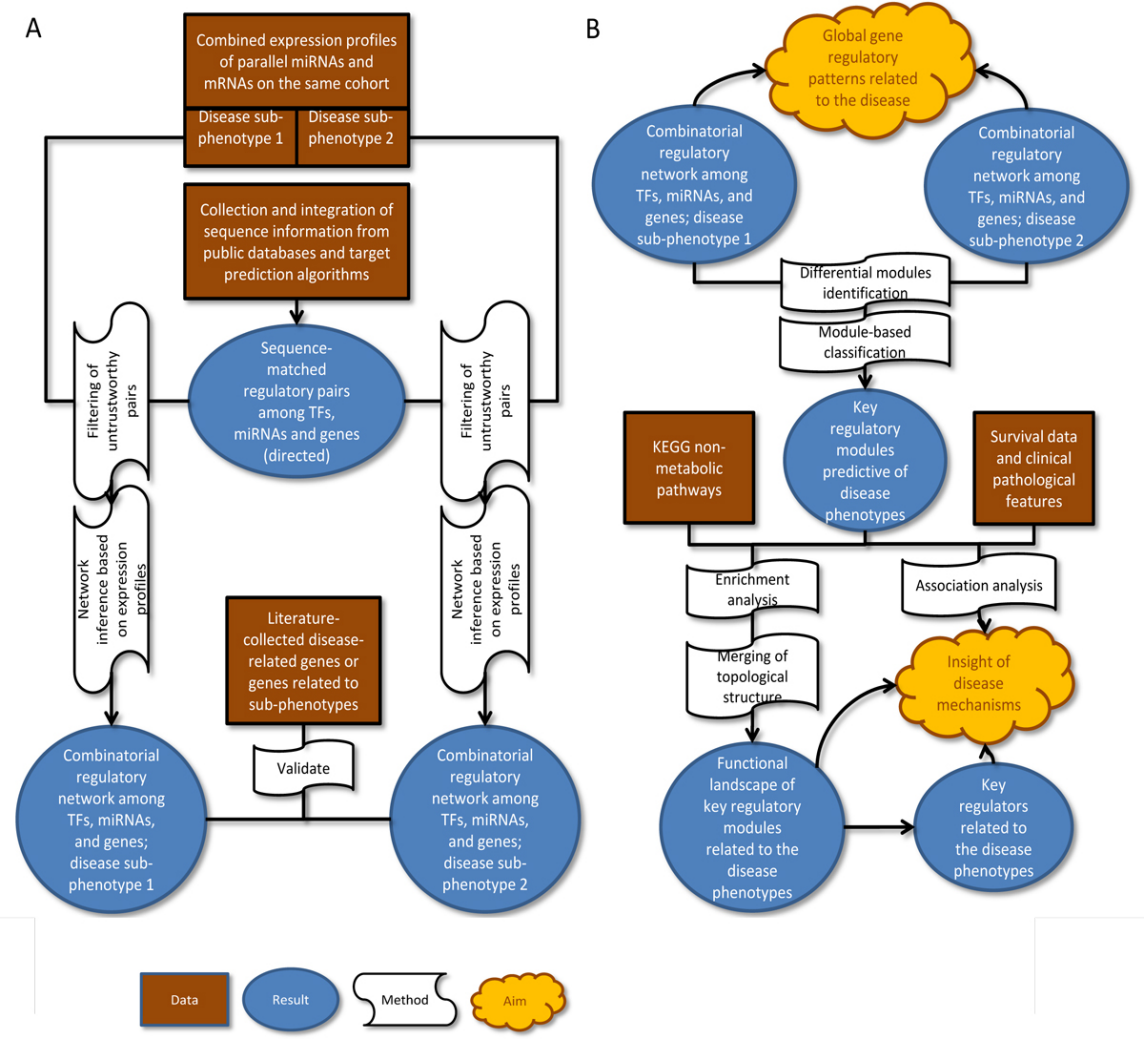
**Schematic overview of constructing combinatorial networks and analyzing differential regulatory modules**. (A). Construction of combinatorial networks. (B). Analyses based on constructed networks.

However, so far neither combinatorial regulatory network analysis nor subnetwork/module marker for risk prediction has been applied under the context of HCC. In this work, to study HCC metastasis, we aim to: 1) investigate global gene regulation patterns involved in HCC progression through combinatorial network analysis, 2) identify key regulatory modules which would not only possess predictive ability for HCC metastasis, but also provide insight of metastasis mechanisms. We selected a set of parallel mRNA and miRNA profiles of HCC patient cohort from a region of endemic HBV infection, and patients were labeled either without or with venous metastasis. The workflow design is illustrated in Figure 1.

As a result, we constructed and compared the TF-miRNA-gene regulatory network in HCC without or with venous metastasis, and thus revealed some molecular characteristics of HCC metastasis. The credibility of the resultant network was estimated by databases and literatures. We identified key regulatory modules that are physically connective and biologically cohesive. The prediction performance for metastasis with our classifying modules was evaluated, which was significantly better than the counterpart gene-list classifiers using leave-one-out cross-validation on the same patient cohort. Some novel key miRNA regulators in HCC metastasis and their mechanisms were implied.

## Results

### Overview of network statistics and validation of the non-metastatic and metastatic HCC networks

We obtained two HCC-related networks corresponding to without- or with- metastasis status. The types of nodes included TF, miRNA, and non-TF-gene; the types of edges included TF-TF, TF-miRNA, TF-gene, miRNA-TF, miRNA-gene. The statistics about nodes and edges are shown in Table 1 in which the regulators included all the nominal TFs or miRNAs in the network, with targets or not. From a global view, the metastatic network was larger and more complex as there are more nodes and edges. We could also see that overall the amount of TFs as nodes and TF-involved edges are both larger than that of miRNAs, no matter in the non-metastatic or metastatic network, which substantiate the critical role of the TF in gene regulation.

**Table 1:** Overall statistics about the nodes and edges of the HCC non-metastatic and metastatic networks

	Non-metastatic	Metastatic
**#Nodes**	**1225**	**1755**
#TF	135	176
#miRNA	20	63
#gene	1070	1516
**#Edges**	**1510**	**2104**
#TF-TF	111	124
#TF-miRNA	2	5
#TF-gene	1350	1761
#miRNA-TF	4	21
#miRNA-gene	43	193

To verify whether our networks are correlated to HCC, we performed one-sided Fisher's exact test respectively on the genes from the two networks and the collected HCC-related genes and HCC-metastasis-related genes resorted from a series of a priori databases and literatures. It turned out that genes from the non-metastatic network were significantly overlapped with HCC-related genes (p = 8.35e-8) but not to HCC-metastasis-related genes (p = 0.094), and that genes from the metastatic network were not only significantly overlapped with HCC-related genes (p = 3.81e-9) but also with HCC-metastasis-related genes (p = 0.031). Such results gave us confidence that our constructed networks reasonably lie in the context of HCC and HCC metastasis.

### Comparison of global regulatory patterns between non-metastatic and metastatic HCC networks

**Figure 2:**
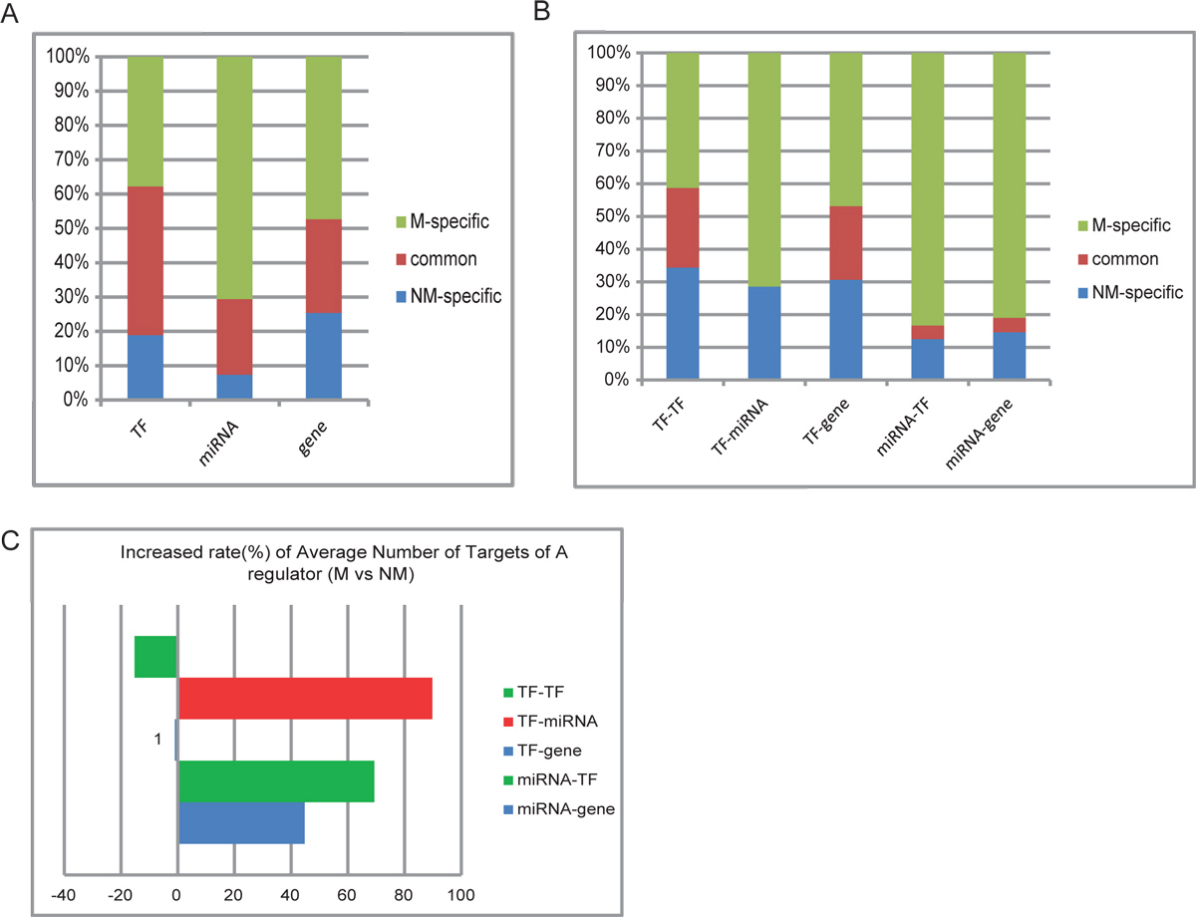
**Comparison of global regulatory patterns between the HCC non-metastatic and metastatic networks**. (A)(B). Comparison of node- and edge- distributions between the HCC metastatic and non-metastatic networks. Nodes or edges were divided into three categories: only in non-metastatic network(NM-specific), only in metastatic network(M-specific), and common in both networks. (C). Increased rate of average number of targets of a regulator in the metastatic network versus the non-metastatic. For each TF- or miRNA- relations as a whole, average number of targets was calculated in each network, and then the increased rate of the average number of targets in the metastatic network versus the non-metastatic one was represented in barplot. The color of the bar represents the type of targets, TF in green, miRNA in red, and gene in blue.

In order to explain the difference of the two networks, we categorized all the nodes and edges into three groups: a) NM-specific nodes or edges that appeared in the non-metastatic network only, b) common nodes or edges that existed in both non-metastatic and metastatic networks, and c) M-specific nodes or edges in metastatic network only. The percentage of each type of nodes and edges in different groups are shown in Figure 2A, B. MiRNAs as nodes appear most in the M-specific group compared with TFs and non-TF genes which appear nearly equally in different groups. MiRNA-involved edges, including TF-miRNA, miRNA-TF, miRNA-gene, also count for an overwhelming proportion in the M-specific group. When considering the increased rate of average number of targets of a regulator in the metastatic network versus the non-metastatic(Figure 2C, Additional File 3), on average a TF regulates more miRNAs but less TFs, and a miRNA regulates both more TFs and genes. These discoveries suggest that miRNAs might participate more actively in tumors with metastasis, which supports the important role of miRNAs in tumor progression [17].

### Identification of key regulatory modules predictive of HCC metastasis

The basic standards on the defining of our key regulatory modules from the combinatorial networks are as follows: i) the selected module should possess clear biological structure to decipher its regulatory pattern. ii) the selected module should contain nodes and edges discriminative of the metastasis status. With such standards, we obtained 71 ranked differential regulatory modules from the two networks in total, each including one specific regulator and all of its first-layer targets, of which 26 were from the non-metastatic network(NM modules) and 45 from the metastatic network(M modules). Based on these differential regulatory modules a series of classification analyses were performed to further identify predictive modules.

Firstly each single module was tested for classification efficiency. Each of the top 20 modules (involving 5 NM- and 15 M- modules) from the ranked differential list was sequentially taken as the single-module classifier and tested in the recursive partitioning classification model [18]. The performance of these single-modules was evaluated by leave-one out cross validation (LOOCV), the best of which achieved accuracy (ACC) of about 82%, and Matthew Correlation coefficient (MCC)of 64%. And there was no significant difference between the performance of modules from the non-metastatic network and the metastatic one(Additional File 3).

**Figure 3:**
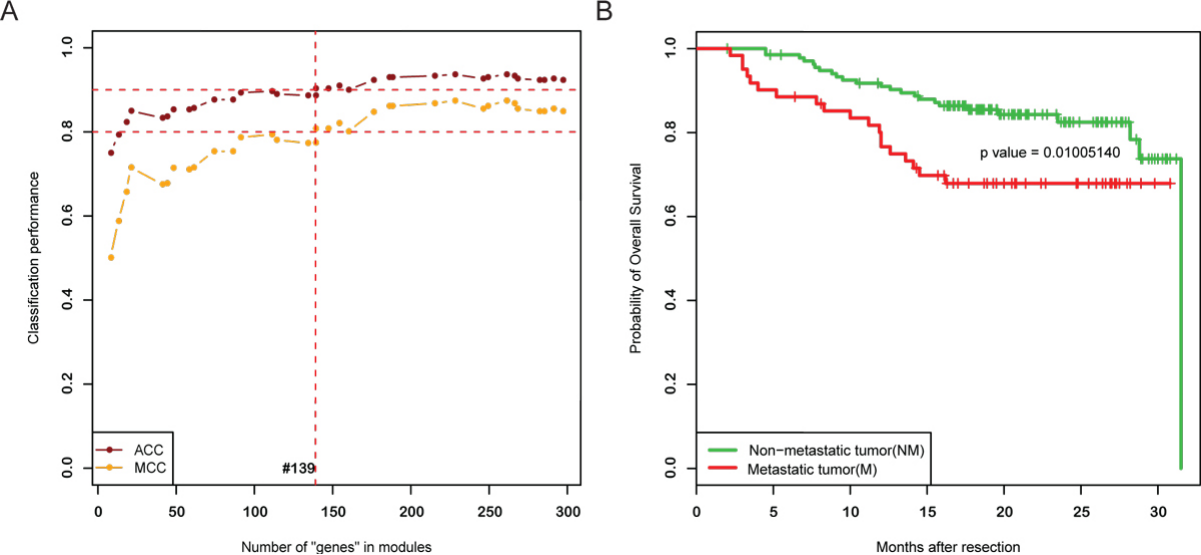
**Performance of the 17-module classifier**. (A). Classification performance of accumulated modules. The x axis represents the number of "genes" involved in the modules, including TFs, miRNAs, or genes, and the y axis represents the value of prediction accuracy(ACC) and Matthew correlation coefficient(MCC). Performance of classifiers whose number of "genes" within 300 are showed. One dot on a line represents addition of one more module. When the number of modules accumulated to 17, ACC overrides 90% and MCC overrides 80%. (B). Survival difference of the predicted non-metastatic and metastatic groups by the 17-module classifier. Kaplan-Meier estimation was calculated to plot the survival curve. Log-rank test was used to compare two survival distributions and generate the p value.

Then cumulative modules were examined for their predictive ability of metastasis status. The classification procedure was repeated by adding one more candidate module at a time from the top down the previously prepared ranked list. Our results showed that when the top 17 modules were chosen as cumulative-module classifier, the LOOCV accuracy overrode 90% and MCC overrode 80% (Figure 3A, Additional File 3), which was an explicitly great improvement than the single-module classifiers. From then on when more modules were added to the classifier, more genes were brought into, while the performance did not improve significantly. So these 17 modules were considered as the key regulatory modules predictive of HCC metastasis in our work, which altogether involved 139 unique genes and miRNAs in 5 NM and 12 M modules. The full list of these modules is displayed in Table 2.

**Table 2:** Full list of 17 regulatory modules predictive of HCC metastasis.

Module name	Regulator	Targets
hsa_miR_326_M	hsa-miR-326	ARHGDIA, CEP250, MYO6, TYR, PWP2, RCBTB2, POLR3F
hsa_miR_323_3p_M	hsa-miR-323-3p	BCLAF1, SUMO1, TMBIM6, FAM168B
hsa_miR_16_M	hsa-miR-16	NFATC3, ETNK1, BMX, NCOR2, POLR3F
hsa_let_7e_M	hsa-let-7e	CLP1, NGF
FOXO3_M	FOXO3	MICAL1, SAMD8, FUBP3, ATXN10, ADAM11, RAB5C, MRPS24, DPAGT1, GPS1, SNRPC, SUMO1, TWF1, SAR1A, PICALM, TXNDC5, HEXIM2, TRIP12, ZDHHC15, SEMA4G, EFHD2
hsa_miR_22_M	hsa-miR-22	SLC6A1, SLC35A4
hsa_miR_326_NM	hsa-miR-326	MTERFD2, ARHGDIA, PCSK4, CEP250, PTRF, MYO6, ST6GALNAC6
hsa_miR_204_M	hsa-miR-204	CHD5, ATF2, POU2F2, TOMM70A, WDR26, SPOP, FAM168B, PLAA, WASF2, SRXN1
POU2F2_M	POU2F2	SPIB, C20ORF43, SUCNR1, PTRF
NFYB_M	NFYB	NTN4, CACNG5, C12ORF10, TUBA1B, CALB2, RGMA, APOC3, PGD, NDUFV1, CHDH, FBXO24, TCTN2
hsa_miR_30a_M	hsa-miR-30a	CREB1, PAWR, NEDD4, RRAS2, VPS26B, TBC1D2B, HTR4, ACAP2, ZFAND5, SPAG9, MICAL1, ATG5
hsa_miR_7_M	hsa-miR-7	PDCD2, POLR2E, NF2, FAM168B, MEGF9
CUX1_NM	CUX1	RUNX1, IFITM2, MARCH5, GPR21, RPL35, TNFRSF10B, CFP, SDHAF2, NUP62CL, YARS, NAGK, GRAMD1A, PLXNB2, BCL2L13, METTL11A, MARK3, ITM2A, HIP1R, BSG
FOXO3_NM	FOXO3	MAFF, LEPROT, MICAL1, PSME1, SAMD8, FUBP3, ATXN10
STAT1_NM	STAT1	MYBL1, MAFF, POLA1, EXOG, PGM1, ZDHHC4, WDR24, AMFR, RAD52, TMEM208, MRPL34, GCHFR, ANKRD30A, TRO, LDHAL6A, SERPING1, RNASE4, ARPC5L, SRSF3, CD248
TP53_NM	TP53	ANKRD52, SLC25A20, PGM1, C1QTNF4, PKDCC
STAT1_M	STAT1	WDR24, RAD52, GCHFR

NM indicates the existence of the module in non-metastatic HCC gene regulatory network, M indicates the existence of the module in metastatic HCC gene regulatory network.

### Comparison of predictive ability of the cumulative modules to gene-list signatures

Some previous reports demonstrated the advantage of subnetwork classification over single gene-lists, probably because of functional relevance in the classifier [11]. In order to check whether our key regulatory modules possess such advantage, we performed gene-list-based classification procedure in a counterpart way to our module-based classification. Signature genes were the selected differentially expressed genes in HCC metastatic vs. non-metastatic samples, with the Student t-test Benjamini-Hochberg adjusted p value < 0.001, and further ranked by the method of minimum redundancy and maximum relevance(MRMR) [19](Additional File 1), which resulted in a list of 349 ranked candidate genes. Same number of genes as in the cumulative-module classifier were picked with priority from the ranked gene list to compose the single-module classifier or perform metastasis classification and the performance was evaluated by LOOCV. Our results showed, there existed no significant difference of ACC or MCC between the top 20 single-module-classifiers and counterpart gene-list-classifiers (two sided t test p value > 0.5) (Additional File 3). However, when combining modules (even just two) the cumulative-module classifier achieved consistently better performance than the classifying models of corresponding number of signature genes (Additional Figure 2, Additional File 3).

### The functional regulatory landscape of the key regulatory modules for HCC metastasis

**Figure 4:**
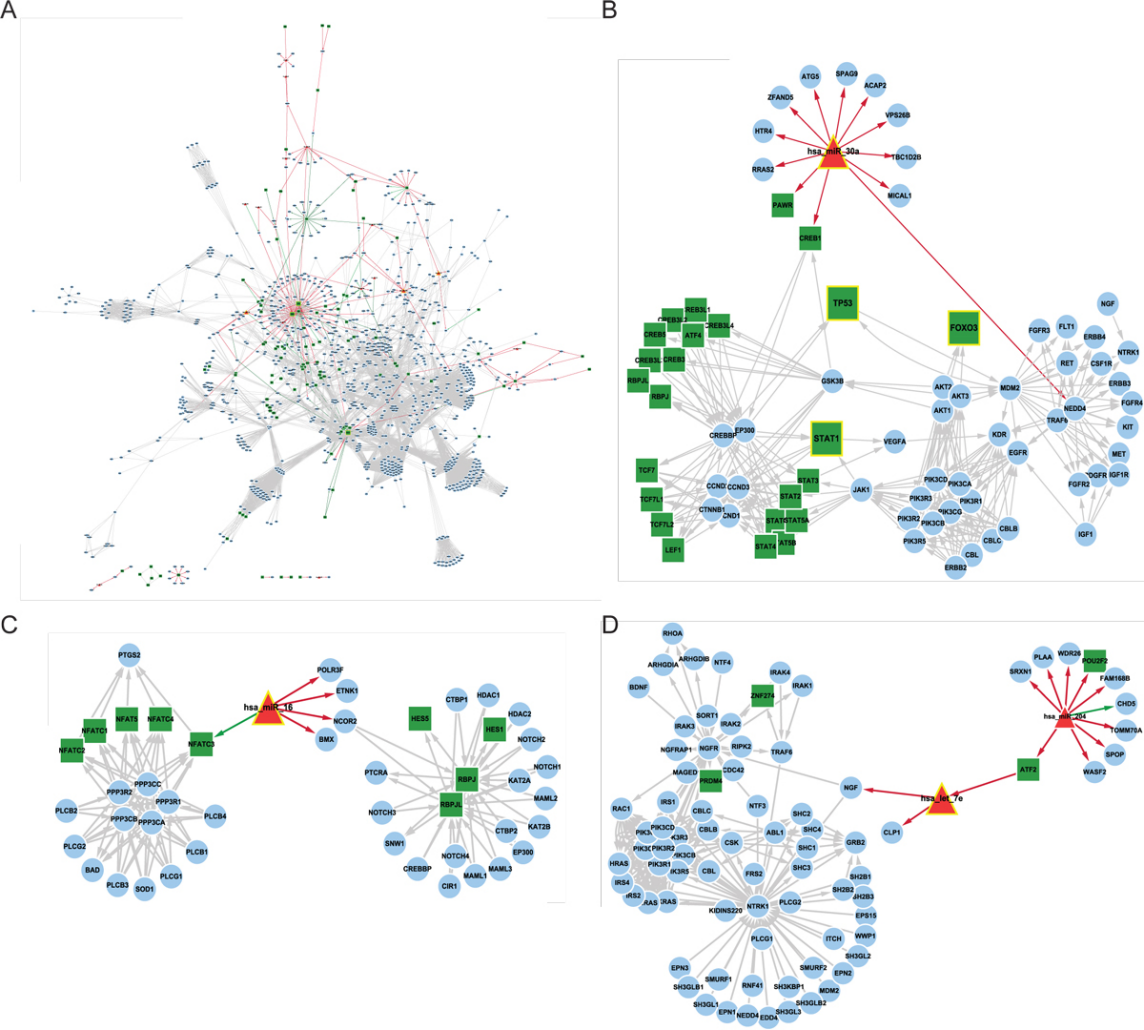
**Differential regulatory network and key miRNA regulators of HCC metastasis**. (A). Differential regulatory network of the 17 classifying modules and their enriched pathways. The green edges represent edges whose CLR weights are larger in network of non-metastasis, while the orange ones represent edges whose weights are larger in network of metastasis. The color and shape of the nodes represent the type of "genes": TF in green rectangle, miRNA in red triangle, and gene in blue eclipse. Six regulators of which modules were enriched in KEGG non-metabolic pathways are highlighted in nodes with larger size and yellow border (hsa-miR-16, hsa-miR-30a, hsa-let-7e, STAT1, TP53, FOXO3). The graph structure of KEGG pathways embodied by gene(protein)-gene(protein) interactions was retrieved by the R package KEGGgraph. (B). Differential regulatory network of miR-30a. (C). Differential regulatory network of miR-16. (D). Differential regulatory network of let-7e/miR-204.

According to the approach of identifying key regulatory modules predictive of metastasis sub-statuses, these modules possess regulatory patterns which were disturbed in tumors with metastasis(either disappeared or appeared). To investigate the disturbed pathways of these NM and M modules, we conducted enrichment analysis on genes from each of the key regulatory modules with all non-metabolic KEGG resource containing gene regulatory and signaling pathways. It turned out that only 6 modules (headed by 6 regulators) out of the 17 were significantly enriched in 28 non-metabolic pathways (Table 3). NM key modules(FOXO3_NM, TP53_NM) were enriched mostly in various cancer pathways and several cancer related processes such as cell cycle and apoptosis; M key modules (hsa_miR_16_M, has_let_7e_M, has_miR_30a_M, STAT1_M) were mostly enriched in signaling pathways related to tumor progression. In order to illustrate a full functional regulatory landscape in the context of HCC metastasis, we merged the topology structure of the 17-module classifier and the enriched 28 non-metabolic KEGG pathways (Figure 4A). The graph structure of KEGG pathways embodied by gene(protein)-gene(protein) interactions was retrieved by the R/Bioconductor package KEGGgraph. In this way, individual key regulators and its module can be zoomed in to see in detail the regulatory pattern transition between non-metastatic status to metastatic status.

**Table 3:** Enriched KEGG non-metabolic pathways of the 17 key regulatory modules.

Module name	KEGG pathway	P value
FOXO3_NM	03050~Proteasome	0.0342
	05213~Endometrial cancer	0.0213
	05223~Non-small cell lung cancer	0.0221
TP53_NM	04110~Cell cycle	0.039
	04115~p53 signaling pathway	0.0212
	04210~Apoptosis	0.027
	04310~Wnt signaling pathway	0.0459
	04722~Neurotrophin signaling pathway	0.0384
	05014~Amyotrophic lateral sclerosis (ALS)	0.0163
	05210~Colorectal cancer	0.0191
	05212~Pancreatic cancer	0.0215
	05213~Endometrial cancer	0.016
	05214~Glioma	0.02
	05215~Prostate cancer	0.0273
	05216~Thyroid cancer	0.009
	05217~Basal cell carcinoma	0.0169
	05218~Melanoma	0.0218
	05219~Bladder cancer	0.013
	05220~Chronic myeloid leukemia	0.0224
	05222~Small cell lung cancer	0.0258
	05223~Non-small cell lung cancer	0.0166
hsa_miR_16_M	04330~Notch signaling pathway	0.024
	04370~VEGF signaling pathway	0.0386
	04662~B cell receptor signaling pathway	0.0381
hsa_let_7e_M	04210~Apoptosis	0.0181
	04722~Neurotrophin signaling pathway	0.0258
hsa_miR_30a_M	04140~Regulation of autophagy	0.0425
	04144~Endocytosis	0.0264
STAT1_M	03440~Homologous recombination	0.0086
	04062~Chemokine signaling pathway	0.0385
	04620~Toll-like receptor signaling pathway	0.0209
	04630~Jak-STAT signaling pathway	0.0317
	05212~Pancreatic cancer	0.0144

One-sided Fisher's Exact Test was used to test whether the genes in a module were significantly enriched in any KEGG non-metabolic pathways. Six modules (FOXO3_NM, TP53_NM, hsa_miR_16_M, hsa_miR_30a_M, hsa_let_7e_M, STAT1_M) of which the resultant p values less than 0.05 are included in this table.

### Key miRNA regulators from the functional landscape of HCC metastasis

Out of 17 key classifying modules predictive of metastasis, six were enriched in KEGG non-metabolic pathways: FOXO3_NM, TP53_NM, STAT1_M, hsa_miR_16_M, has_let_7e_M, has_miR_30a_M. Based on the hypothesis that miRNAs might actively participate in the tumor progression and metastasis process and act as key roles, we further focused on the regulatory patterns of the three modules headed by miRNAs, which were zoomed in from the regulatory landscape constructed above.

MiR-30a. The module led by miR-30a in the metastatic network shows inextricable links to various cancer-related pathways and some other important regulators (Figure 4B). EP300 and CREBBP, regulated by way of miR-30a and CREB1, are highly related transcriptional co-activators possessing histone acetyltransferase activity and were known to be involved in the survival and invasion pathways of prostate cancer [19]. Functions of TP53 and STAT1 might be modulated through acetylation by CREBBP/EP300. Meanwhile, NEDD4, another target of miR-30a, by further targeting EGFR, might interfere with key cellular signaling pathways. According to a previous report, miR-30a was reported to inhibit epithelial-to-mesenchymal transition in non-small cell lung cancer [20]. The exact role miR-30a might play in HCC metastasis requires more exploration.

MiR-16. MiR-16 targets human nuclear co-repressor 2(NCOR2) in the metastatic network. NCOR2 is a transcriptional co-repressor linked to Notch (Figure 4C). According to previous reports, Notch signaling cascade was regarded as anti-proliferative rather than oncogenic in hepatocellular carcinoma [21,22], so if miR-16 repressed Notch it might result in more aggressive HCC that would lead to metastasis.

Let-7e and miR-204. In tumor with later metastasis, let-7e targets nerve growth factor (NGF), whose deprivation was supposed to induce apoptosis [23]. Upstream let-7e is ATF2 and miR-204 (Figure 4D). Because we only performed the first-step targets enrichment in KEGG pathways, miR-204 was not among the six key regulators whose targets showed pathway enrichment, yet it was one of the heading regulators of 17 key regulatory modules. Besides, referring to the topology of our HCC metastatic network, miR-204 is a bottleneck with the 7th highest betweenness, and two of miR-204-involved edges rank into the top 10 list of edge betweenness(Additional File 3). Therefore we may hypothesize that the important role of let-7e regulation was driven by its upstream regulator miR-204. MiR-204 represses the expression of its target ATF2, blocking its activation to downstream target let-7e. The lack of let-7e may release NGF deprivation and therefore inhibit apoptosis, leading to tumor aggression and HCC metastasis. Indeed miR-204 was previously reported to regulate mesenchymal progenitor cell differentiation [24]and to be related to head and neck tumor metastasis [25]. Therefore we list both let-7e and its upstream miR-204 to be key regulatory miRNAs that might relate to HCC metastasis.

### Prognosis prediction by the HCC metastasis classifying modules

With the 17 key regulatory modules, all the HCC patients could be classified into two groups: without or with venous metastasis. To investigate the survival outcomes of these two groups, Kaplan-Meier analysis was performed, and the survival difference between the two groups was evaluated by log-rank test (p = 0.01) (Figure 3B). It turned out that the predicted NM group correlated significantly with a longer overall survival, whereas the M group correlated significantly with a shorter one. The difference of clinical characteristics between predicted groups of venous metastasis were also investigated (Table 4). BCLC stage, AFP level, TNM stage, each showed significant difference between these two groups. As we know, AFP is a common critical index in HCC progression, and BCLC and TNM stages reflect the progression stage of hepatocellular carcinoma. These results suggested that the predicted HCC subgroups of venous metastasis might represent distinct prognosis and clinical stages, which on the other hand subordinate the rational performance of the identified 17 key regulatory modules.

**Table 4:** Comparison of clinical characteristics between predicted subgroups of venous metastasis

	Predicted NM	Predicted M	P value
Patient cohort (n = 198)	n = 137	n = 61	
Gender(male/female)	121/16	53/8	0.9601
Age(yr, mean ± SD)	49.99 ± 11.22	50.15 ± 9.54	0.8235
Number of nodule(1/2/3/4)	117/18/2	55/4/1/1	0.2603
Tumor capsule(complete/none)	53/84	24/37	0.9441
Cirrhosis(no/yes)	8/129	5/56	0.7584
AFP(log2-transformed, mean ± SD)	6.79 ± 3.96	8.08 ± 4.56	**0.0395**
TB(μmol/L, median(25-75%))	15.4(12.1-20.2)	17.4(11.4-22.1)	0.2954
ALT(μ/L, median(25-75%))	43(31-61)	49(32-66)	0.3929
OKUDA stage(0/1)	119/18	49/12	0.3325
CLIP stage(0/1/2/3/4)	64/49/22/1/1	28/15/12/4/2	0.0509
BCLC stage(0/A/B/C)	15/103/14/5	7/35/7/12	**0.0022**
TNM stage(I/II/III)	65/57/15	23/23/15	**0.0441**
Child-Pugh class(A/B)	132/5	57/4	0.5910

P value: Comparison between clinic pathological indicators of non-metastatic and metastatic groups was conducted using chi-square test for discrete variables and Wilcoxon test for continuous variables.

**Figure 5:**
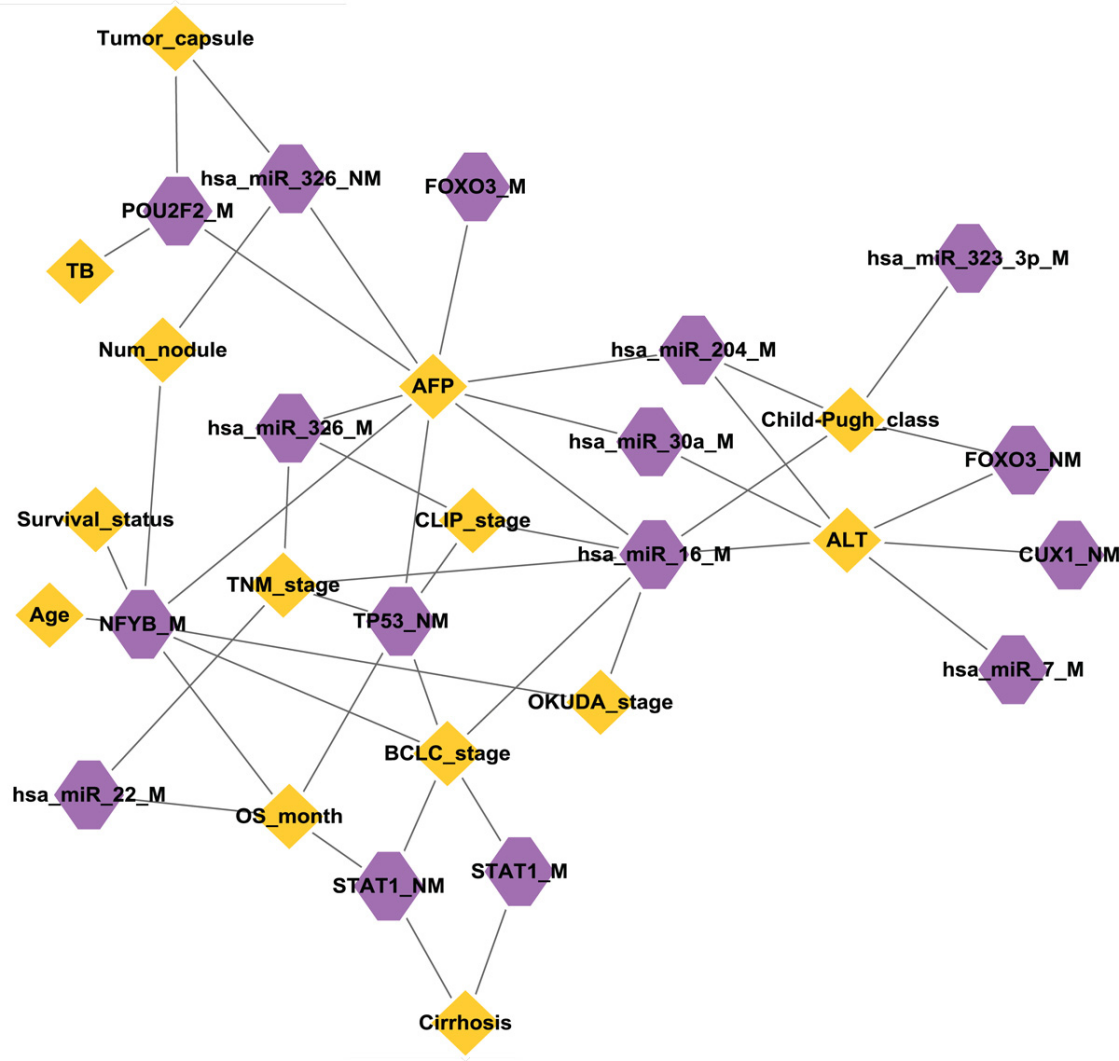
**Association network of key regulatory modules to clinical pathological characteristics**. The association between each key regulatory module and clinical pathologic characteristics was examined using an R/Bioconductor package GlobalAncova. Yellow diamonds represent clinical characteristics and purple octagons represent regulatory modules. An edge connects a module and a clinical parameter if the p value resulted from the GlobalAncova test is significant (p < 0.05). Module hsa_let_7e_M did not have association with any clinical features, so it is not shown in the figure.

Next we scanned the association of each module to clinical features using GlobalAncova (Figure 5). Among the clinical parameters, AFP related with most modules, followed by another clinical parameter, ALT, and then BCLC stage, TNM stage and Child-Pugh class. To be noted, the three modules led by the key regulatory miRNAs identified above (miR-16, miR-30a, miR-204/let-7e) were all closely associated with AFP and ALT. Moreover, hsa_miR_16_M was simultaneously related with the most number of clinical characteristics (AFP, ALT, and 5 cancer staging indexes), which further suggested that miR-16 and its targets might correlate with invasive tumor cell behavior.

## Discussion

By utilizing both sequence information and parallel miRNA and mRNA expression data on the same cohort of HBV related HCC patients, we constructed gene regulatory networks combining TF and miRNA regulation and specific for HCC without or with metastasis. Based on our combinatorial differential networks, global properties of the gene regulatory patterns in different metastasis subgroups were analyzed. TFs accounted for the main part of regulation, miRNAs for the minor part of regulation; miRNAs played a more active role in the metastatic network. Then differential regulatory modules discriminative of the metastatic status were extracted, and module-based classifier for metastasis prediction was constructed. Module-based classifier achieved better classification performance than the differential gene list-based classifiers. Furthermore, a few novel potential metastasis-related key miRNA regulators were proposed, such as miR-16, miR-30a, and let-7e/miR-204. Biological implications and differential regulatory patterns of key miRNAs were examined through functional regulatory landscape. Survival analysis and clinical characteristics association were conducted to support the importance of the classifying modules and the key regulators.

It is generally conceived that in transcriptional regulation TFs play the controlling roles and miRNAs make auxiliary contributions [13]. In our work, we concordantly got that TFs made up the main part of nodes and edges in HCC networks without or with metastasis, and miRNAs participated in fewer regulations than TFs. However, we also found that miRNAs showed an increased amount of regulations in the metastatic network. Judged by the basic topological properties such as degree, betweenness, and edge betweenness (Additional File 3), most hubs and bottlenecks in both networks were TFs, but one miRNA, hsa-miR-204, was listed as the 7th bottleneck in the metastasis network according to its betweenness. All the top 10 edges with the largest edge betweenness involved only TFs in the non-metastasis network, but 4 out of 10 top edge-betweenness edges in the metastasis network involved miRNAs. Besides, TFs in the metastatic network tend to regulate genes by way of miRNAs (Figure 2C). It might be implied from our results that the process of tumor progression and metastasis is complicated and delicate therefore it takes up more auxiliary regulatory functions performed by miRNAs, in order to facilitate broader regulations by TFs.

The 17 key classifying modules identified in this work were not merely sub-networks, but 'regulatory' modules, each defined as a regulator and its first layer target genes. All the classifying regulatory modules possessed distinct regulatory patterns in either non-metastatic or metastatic subgroup. Since Chuang et.al proposed a pivot method for network-based classification in 2007 [11], various alternative methods based on network modules have been reported [26,27]. The 17 key regulatory modules in this work could nicely classify patients into different metastasis sub-groups. Six modules' regulatory targets could be enriched in KEGG non-metabolic pathways, allowing an even clearer elucidation of their functional regulation patterns. It is conceivable that such differential regulatory modules discriminative of metastasis sub-groups might better imply the mechanisms of tumor progression and invasion. The regulatory landscape we drew for these modules could be zoomed in to check in detail the possible roles of each interested module or regulator played in HCC metastasis. We exemplified such analyses by looking at three microRNA modules whose target gene members were enriched in KEGG pathways: miR-30a, miR-16 and let-7e modules. Let-7e module is connected to miR-204, which is another key regulator in metastatic network.

Metastasis is known to be a sign of higher grade of malignance and undermine survival time. The fact that the predicted metastatic group had a significantly worse prognosis in survival analysis justified the classification performance of the selected 17 modules. The predicted metastatic group patients also showed worse BCLC staging, TNM staging, and higher alpha-fetoprotein(AFP) values compared to non-metastatic group. Increased AFP value is a long-established factor of HCC progression. Furthermore, modules headed by the three key miRNAs were associated with both AFP and alanine aminotransferase(ALT), implying that ALT value might also be closely related to venous metastasis in HCC. Module hsa_miR_16_M was simultaneously significantly associated with five cancer staging systems, which further supported the key role of miR-16 in HCC metastasis.

Compared with our preliminary work in 2009 [16], improvements were achieved not only in biological interpretation but also in network inference algorithm. The linear regression modeling approach we used in 2009 had a shortcoming in that it attempted to determine the regulation structure for each target gene independently, while it is well known that genes that share the same expression pattern are likely to be involved in the same regulatory process, and therefore share the same (or at least a similar) set of regulators. In this work, we used mutual information metric that detects statistical dependence between two variables with no assumption of linearity of the dependence. Among the various gene network inference algorithms based on mutual information developed by different groups such as RN [28], ARACNE [29], CLR [30], MRNET [31], CLR resulted in the highest true positive rate compared with the others according to a previous report [32]. Therefore CLR algorithm was selected for network inference for our work, as we required all the edges in our network to be also sequence-matched besides expression-correlated, reducing the false positive rate of expression-inferred edges.

The workflow in our study is not restricted in this study alone. According to the schematic illustration (Figure 1), researchers may conduct similar analysis given the data (depicted as rectangle) available for the context. In practical terms, if parallel miRNA and mRNA expression profiles are available on the same cohort of patients with known disease phenotypes, the workflow in this study can be extended to other biomedical problem or cancer context by integrating data from public databases or literatures. The major programs in the workflow were either self-written scripts with little programming complexity or open-source R/Bioconductor packages which were confirmed to be useful and efficient in this study. As to compute runtime and complexity, the most time-consuming step in our workflow was in the network inference, because the CLR algorithm has a complexity in O(*n*2*p*2) since all pairwise interactions are considered [33]. It computes the mutual information(MI) matrix first, transforms the MI matrix into scores that take into account the empirical distribution of the MI values, and then applies a threshold. When the expression values of genes are treated as continuous random variables and the MI is estimated by kernel methods, computing the pairwise MI can be computationally expensive. In our study, dimension reduction was conducted(filtering of untrustworthy pairs) before the CLR network inference so as to cut down the computational *complexity *and complete the computation within the *limits *of system memory.

In summary, in this work we demonstrated an integrative approach to conduct differential combinatorial regulatory network analysis in the specific context of HCC metastasis. Through this systematic analysis, we proposed changes of global regulatory patterns in HCC progression, and identified some key miRNA regulators contributed to HCC metastasis whose regulatory patterns and biological implication were also deduced. Before this, although multi-perspective data have been integrated into HCC-related analyses [6-8], no peer works providing global landscape of combinatorial gene regulatory network or identifying module classifiers for risk prediction has ever been reported in the specific context of venous metastasis of HBV-HCC. Our results proposed possible transcriptional regulatory patterns underlying the different metastatic subgroups of HCC. Meanwhile, miR-30a and miR-16, let-7e/miR-204, which had not been taken as granted to be related with metastasis, especially in HCC, stood out from our results, which may merit further experimental validation. Our results might facilitate the understanding of the molecular regulatory mechanisms and role of miRNAs in HCC metastasis. The workflow in this study can also be applied in similar context of cancer research or extended to other topics.

## Conclusions

In this work we demonstrated an integrative approach to conduct differential combinatorial regulatory network analysis in the specific context of HCC metastasis. Through this systematic analysis, we proposed changes of global regulatory patterns in HCC progression, and identified some key miRNA regulators contributed to HCC metastasis whose regulatory patterns and biological implication were also deduced. Our results might facilitate the understanding of the molecular regulatory mechanisms and role of miRNAs in HCC metastasis. The workflow in this study can also be applied in similar context of cancer research or extended to other topics.

## Methods

### Datasets and patients

mRNA and miRNA expression microarray data on the same cohort of HBV-infected HCC patients who underwent radical resection in Zhongshan Hospital were used for integration in this study. Both datasets (GSE5975 and GSE6857) were downloaded from the Gene Expression Omnibus (GEO) database http://www.ncbi.nlm.nih.gov/geo/. The mRNA signal intensities were retrieved from GSE5975, which was generated using the NCI/ATC Hs-OperonV2 array. The miRNA expression levels were obtained from GSE6857, which was generated using OSU-CCC MicroRNA Microarray Version 2.0. Status of venous metastasis of patients were collected from GSE6857. Other clinical pathologic characteristics and survival time of patients were provided by Zhongshan Hospital.

### Data preprocessing for combined expression

Microarray data preprocessing was conducted on each dataset separately, and then both mRNA profiles and miRNA profiles were combined to prepare the combined expression profiles among the 198 patients, 150 non-metastatic and 48 metastatic. After quantile normalization across arrays on the combined expression profiles, irrelevant genes and mature-miRs within the 5% smallest standard deviations of tumor/nontumor profiles between metastasis and non-metastasis samples were filtered. Finally, the combined mRNA and miRNA expression profiles of 198 patients included 12434 genes and 132 mature-miRs altogether. More detailed information for data processing is available in Additional File 1.

### Candidate sequence-matched relationships between TFs, miRNAs, and genes

In the following data selection, a gene list of 1318 previously defined TFs [34] from a previous report were regarded as TFs, while others as non-TF genes.

MiRNA-gene. Candidate miRNA-target relationships were downloaded from miRBase Target Version 5.0, TargetScanHuman Version 5.1, and miRDB Version 3.0, each was based on the predicting algorithm miRanda [35], TargetScan [36], and miRTarget2 [37], respectively. The predicted miRNA-gene relationships with accordance in at least two algorithms were retained in our study.

TF-gene. A set of predicted TF-gene relationships were compiled with methods mainly described in our previous work [16], where TF-TFBS(TF binding sites) relationships and TFBS-gene relationships were first calculated, based on which TF-gene relationships were linked. The difference between the method in this work and our previous work was that the promoter region of each gene in our work was defined as 1k bp up- and down- stream (instead of 1 kb upstream to 0.5 kb downstream of the transcription start site (TSS) according to the ENCODE project [38].

TF-miRNA. TFBSs mapped to the regions upstream of miRNA primary transcript TSSs were downloaded from miRGen 2.0 [39]. Precursor-miRs were mapped to mature-miRs according to miRBase database. Then the candidate TF-miRNA relationships were generated based on the above TF-TFBS relationships and TFBS-miRNA relationships.

The statistics of the final set of 327711 regulatory relationships based on sequence-matched in human between TFs, miRNAs, and genes were displayed in Additional File 3.

### Fisher Exact test to compare constructed networks with HCC-related and HCC-metastasis-related genes

HCC-related genes were collected from HCCdb [40], EHCO-II [41], and HCCNet [42]. The union set of 5088 genes from these three databases was taken as the HCC-related genes. HCC-metastasis-related genes were collected using the text-mining tool, SciMiner [43]. ("carcinoma, hepatocellular"[MeSH Terms] OR hepatocellular carcinoma[Text Word]) AND ("liver neoplasms"[MeSH Terms] OR liver cancer[Text Word]) AND ("neoplasm metastasis"[MeSH Terms] OR metastasis[Text Word]) AND metastatic[Text Word]) was set as the query string for full text mining. The resultant 322 genes each cited by at least 2 papers were regarded as the HCC-metastasis-related genes in this study. All the collected HCC-related genes and HCC-metastasis-related genes are listed in the Additional File 4.

One sided Fisher's Exact Test was performed to examine whether genes in our constructed HCC non-metastatic and metastatic networks were significantly overlapped with the collected HCC- or HCC-metastasis- related genes from databases and literatures. All the 12434 genes in the combined expression profiles were used as the set of universe genes in the test.

### Network construction

The combined expression profile was divided into two sub-profiles by sample labels, namely profile of non-metastasis and profile of metastasis, so as to construct gene regulatory network of HCC without and with metastasis respectively.

We assumed that sequence-matched pairs were more possible to be real interaction pairs than sequence-unmatched pairs, and that real interaction pairs were more possible to be correlated in expression than random pairs. In order to construct the network as credible as possible, we filtered out untrustworthy pairs before expression-based network inference. The candidate 327711 sequence-matched relationships genome-wide were first reduced to 78310 non-self-looping pairs whose both nodes were genes and miRNAs with expression in the combined profiles. Then the absolute spearman correlation of the expression was calculated between each of these 78310 sequence-matched pairs, and the mean absolute spearman correlations of the expression were also calculated between randomly sampled 78310 pairs from the combined expression profile for 100 random times. Pairs with the absolute spearman correlation higher than 95% of random pairs were retained as candidate pairs, which were processed to infer the transcriptional interactions.

Based on the two sub-profiles respectively, based on all nodes from the above remaining pairs, Context Likelihood of Relatedness (CLR) [30] was then applied as the network inference algorithm to identify transcriptional interactions using an R/Bioconductor package minet with default parameters. The CLR algorithm returned a non-negative matrix which was the weighted adjacency matrix of the network whose values represented the edge weights of the network. We set the cutoff for edge weights as 1, and edges whose edge weight below 1 were thus removed, since edges with little weight were considered as marginal relationships and might be noise.

CLR uses mutual information as a metric of similarity between the expression profiles of two genes. Formally, the mutual information for two discrete random variables X and Y is defined as:

(1) $$I\left(X;Y\right)=\underset{i,j}{\sum }p\left(x_{i},y_{j}\right)\text{log}\frac{p\left(x_{i},y_{j}\right)}{p\left(x_{i}\right)p\left(y_{j}\right)}$$

where *p*(*x_i_*, *y_j_*) is the joint probability distribution function of *X *and *Y*, and *p*(*x_i_*) and *p*(*y_j_*) are the marginal probability distribution functions of *X *and *Y *respectively. In the case of continuous random variables, the summations over *X *and *Y *are replaced by integrals. For genes, *X *and *Y *represent a transcription factor and its potential target gene, and *x_i _*and *y_i _*represent particular expression levels (Further description in Additional File 1).

### Classification of metastasis based on gene regulatory modules

The composition of our 'modules' was defined as one specific regulator and all of its first-layer targets (more than one), and was named as Regulator_Status. Regulator was the name of the regulator, i.e. a TF or a miRNA. Status represented the source network of the module; it could be from the non-metastatic or metastatic network. All the modules in our work included targets only one step down from the regulator such that the regulatory attributes of each module was explicit to read.

Differential modules were first selected before identifying predictive classifying modules of metastasis sub-statuses. As to edges, the non-discriminative edges were excluded from the networks. For all the edges appearing in any of the two networks, we calculated the absolute value of the edge weight difference (The edge weights were directly carried on from the CLR results. The edge weight of a non-existing edge was regarded as zero.) between the two sub-statuses. The edges whose absolute edge weight difference were within the lowest 25% among all the edges were regarded as non-discriminative ones and were filtered out. As to nodes, GlobalAncova [44] test was performed on each module to measure the discriminance of nodes in that module between the metastasis statuses, which was implemented using R/Bioconductor package GlobalAncova (Additional File 1). Significant differential modules with Benjamini-Hochberg adjusted p < 0.001 were taken as candidate predictive modules, which were sorted by their corresponding p values from smallest to the largest. Finally, these ranked differential modules were proceeded to classification.

A multivariate algorithm, recursive partitioning, was chosen as the classification model [45]. It creates a decision tree that strives to correctly classify members of the patients based on several dichotomous dependent variables, which is simple and intuitive (Further description in Additional File 1). Recursive partitioning has been successfully applied in other cancer biology context to identify multi-gene biomarkers or signatures [46-48]. In this study, the classification procedure was performed using R/Bioconductor package rpart with default setting of parameters. The predicted group and the prediction possibility for each individual were returned at each performance using this program. For cumulative modules as one classifier (a list of modules), the final predicted label for each individual was determined as the label with the larger overall predicted probability by modules in the classifier; for single-module as one classifier (a list of genes), the final predicted label for each individual was determined as the label with the larger predicted probability. Leave-one-out cross-validation (LOOCV) was used to evaluate the classification performance.

### Clinical association and survival analysis

The survival analysis was performed to compare patient overall survival. Kaplan-Meier estimation was calculated to plot the survival curve. Log-rank test was used to compare two survival distributions and generate the p value. Comparison between clinical pathological indicators was conducted using chi-square test for discrete variable and Wilcoxon test for continuous variables. The association between clinical pathologic characteristics and classifying modules was examined using GlobalAnova test by R/Bioconductor package GlobalAncova (Additional File 1).

## Competing interests

The authors declare that they have no competing interests.

## Authors' contributions

LYZ, YXL and LX conceived and designed the project. LYZ performed major part of the designed study. JY participated in clinical feature association analysis. TH participated in network analysis. FH and WLY participated in selection of HCC metastasis related genes. HLJ, QZD and LXQ provided clinical follow-up information of the patient cohort. LYZ wrote the manuscript. LX revised the manuscript.

## Supplementary Material

Additional file 3 **Supplementary Tables**. Additional Table 1 - Performance of various classifiers. Additional Table 2 - Statistics about average number of targets regulated by TFs and miRNAs. Additional Table 3 - Basic topological properties of the HCC non-metastatic and metastatic networks. Additional Table 4 - Statistics of the total set of sequence-matched pairs. Click here for file

Additional file 1 **Supplementary Methods**. Click here for file

Additional file 4 **Collection of HCC-related genes and HCC-metastasis-related genes**. Additional Table 5 - HCC-related genes. Additional Table 6 - HCC-metastasis-related genes. Click here for file

Additional file 2 **Performance comparison between module-based and gene-list-based classifiers**. Click here for file
