# Supplementary material for: Differential combinatorial regulatory network analysis related to venous metastasis of hepatocellular carcinoma
Source: BMC Genomics. 2012 Dec 17;13(Suppl 8):S14. doi: 10.1186/1471-2164-13-S8-S14 (PMC3535701; doi:10.1186/1471-2164-13-S8-S14)
Supplement: Additional file 1 — Supplementary Methods. [file 1471-2164-13-S8-S14-S1.pdf]

Microarray data preprocessing

The mRNA signal intensities were retrieved from GSE5975, a dual-channel microarray dataset, where cy5 represented tumor and cy3 non-tumor. Data table of each slide in GSE5975 were downloaded from GEO. After removing signals flag tagged or with intensity below 10 and all values log2 transformed, missing values were imputed by K-NN method using the R/Bioconductor package *impute*. The background corrected median intensities were normalized using Lowess normalization. Probe identifiers were mapped to official gene symbols based on the mapping provided by the platform annotation from GEO and by the HUGO Nomenclature Committee (HGNC), and those mapped to the same gene symbol were merged in value through Tukey's Biweight Robust Mean.

The miRNA expression levels were obtained from GSE6857, a single-channel microarray dataset including paired tumor and non-tumor samples. Series matrix of GSE6857 was downloaded from GEO. Probes with flagged values in >20% of arrays were filtered and K-NN imputation was applied to all log2-transformed signal intensities. Lowess normalization was performed array-by-array as the way done in dual channel mRNA microarray where cy5 represented tumor and cy3 non-tumor. Probe identifiers were mapped to pre-miR names by the microarray platform annotation and corresponding ma-miR names by miRBase (www.mirbase.org), and those mapped to the same ma-miR name were merged in value through Tukey's Biweight Robust Mean.

We supposed that if a gene was closely related with the disease progression, its expression profile should show consistent trend in samples from non-tumor to non-metastatic tumor, and to metastatic tumor. In this way, if a gene was closely related with the cancer progression, there should lay a difference of its expression between the ratio of *metastatic tumor/non-tumor* and *non-metastatic tumor/non-tumor*. For this consideration, after quantile normalization was performed across arrays, the 5% of genes with the smallest standard deviations of profile were considered to have invariant ratio of tumor/non-tumor between metastasis and non-metastasis, and were thus filtered for each dataset.

The Context Likelihood of Relatedness network inference algorithm

The CLR algorithm was proposed in 2007 by Jeremiah Faith et.al[1]. It uses mutual information as a metric of similarity between the expression profiles of two genes. Formally, the mutual information for two discrete random variables X and Y is defined as:

$$I(X;Y) = \sum_{i,j} p(x_i, y_j) \log \frac{p(x_i, y_j)}{p(x_i) p(y_j)} \qquad (1)$$

where $p(x_i, y_j)$ is the joint probability distribution function of X and Y, and $p(x_i)$ and $p(y_j)$ are the marginal probability distribution functions of X and Y respectively. In the case of continuous random variables, the summations over X and Y are replaced by integrals. For genes, X and Y represent a transcription factor and its potential target gene, and x_i and y_i represent particular expression levels. Mutual information possesses the flexibility to detect regulatory interactions

that might be missed by linear measures. The CLR algorithm applies an adaptive background correction step to eliminate false correlations and indirect influences. After computing the mutual information between a regulator and a potential target, the algorithm calculates the statistical likelihood of this mutual information value within its network context by comparing it to the “background” distribution of mutual information scores for all possible regulator-target pairs that include either the regulator or its target. The most probable interactions are those whose mutual information scores stand significantly above the background distribution of mutual information scores. Mutual information possesses the flexibility to detect regulatory interactions that might be missed by linear measures.

Recursive Partitioning classification model

Recursive partitioning is a technique that builds tree-based classification rules to predict the class membership on the basis of feature information[2]. It has been successfully applied in biomedical context to identify multi-gene biomarkers or signatures[3][4][5].That is, it creates a decision tree that strives to correctly classify members of the individuals based on several dichotomous dependent variables. At each step, the recursive partitioning program determines for each feature (in this study for each candidate module/candidate gene) a cutoff point that best splits all of the individuals into different (in our study non-metastatic and metastatic) groups and selects the variable that performs best. Next, it takes the resulting sub-groups and repeats the process until either a sub-group contains one class of individuals or the

sub-group is too small to subdivide. It is a nonparametric method in nature which does not make distribution assumptions for the predictor variables (features).

GlobalAncova

GlobalANCOVA is a general methodology for analyzing gene expression data in terms of predefined gene sets, pathways or complexes. Its constructive idea is to use gene-wise linear models and to aggregate their information in a multivariate test procedure[6].

Gene-wise linear models are used to formalize the relationship of gene expression with phenotypic or genomics covariates. An ANOVA-based sum of squares summarizes the individual gene-wise linear models to a group statement. A permutation test and an asymptotic distribution of the test statistics under the null hypothesis are available to calculate P-values. Basically, it allows for testing whether a specific aspect of the study design is necessary to explain the observed gene expression. It is not confined to two-group comparisons, but considers a broader range of designs by exploiting the full scope of linear model theory.

In network module analysis, it has been used to improve the functional interpretation of gene groups by including additional information (e.g. experimental conditions and biological context) associated with the group[7]. And it has also been used to test for the association between expression values and clinical covariates[8].

Maximum Relevance Minimum Redundancy (mRMR) feature selection

The mRMR method was developed by Peng et al[9] in 2005. It ranks each feature according to both its relevance to the target classification variable and the redundancy between the features. A “good” feature is characterized by maximum relevance with the target variable and minimum redundancy within the features. Both relevance and redundancy are defined by mutual information, which estimates how much one vector is related to another. It has been applied in high-dimensional feature selection processes to identify top features for classification[10][11].

Reference:

1. Faith JJ, Hayete B, Thaden JT, Mogno I, Wierzbowski J, Cottarel G, Kasif S, Collins JJ, Gardner TS: **Large-Scale Mapping and Validation of Escherichia coli Transcriptional Regulation from a Compendium of Expression Profiles**. *PLoS Biol* 2007, **5**:e8.

2. Zhang H, Yu C-Y, Singer B, Xiong M: **Recursive Partitioning for Tumor Classification with Gene Expression Microarray Data**. *PNAS* 2001, **98**:6730–6735.

3. Koziol JA, Zhang J-Y, Casiano CA, Peng X-X, Shi F-D, Feng AC, Chan EKL, Tan EM: **Recursive partitioning as an approach to selection of immune markers for tumor diagnosis**. *Clin. Cancer Res.* 2003, **9**:5120–5126.

4. Chen H-Y, Yu S-L, Chen C-H, Chang G-C, Chen C-Y, Yuan A, Cheng C-L, Wang C-H, Terng H-J, Kao S-F, Chan W-K, Li H-N, Liu C-C, Singh S, Chen WJ, Chen JJW, Yang P-C: **A five-gene signature and clinical outcome in non-small-cell lung cancer**. *N. Engl. J. Med.* 2007, **356**:11–20.

5. Jeong Y, Xie Y, Xiao G, Behrens C, Girard L, Wistuba II, Minna JD, Mangelsdorf DJ: **Nuclear Receptor Expression Defines a Set of Prognostic Biomarkers for Lung Cancer**. *PLoS Med* 2010, **7**:e1000378.

6. Hummel M, Meister R, Mansmann U: **GlobalANCOVA: exploration and assessment of gene group effects**. *Bioinformatics* 2008, **24**:78 –85.

7. Wang X, Dalkic E, Wu M, Chan C: **Gene module level analysis: identification to networks and dynamics**. *Current Opinion in Biotechnology* 2008, **19**:482–491.

8. Culhane AC, Quackenbush J: **Confounding Effects in “A Six-Gene Signature Predicting Breast Cancer Lung Metastasis”**. *Cancer Res* 2009, **69**:7480–7485.

9. Peng H, Long F, Ding C: **Feature Selection Based on Mutual Information: Criteria of Max-Dependency, Max-Relevance, and Min-Redundancy**. *IEEE Transactions on Pattern Analysis and Machine Intelligence* 2005, **27**:1226–1238.

10. Huang T, Cui W, Hu L, Feng K, Li Y-X, Cai Y-D: **Prediction of Pharmacological and Xenobiotic Responses to Drugs Based on Time Course Gene Expression Profiles**. *PLoS ONE* 2009, **4**:e8126.

11. Huang T, Wang P, Ye Z-Q, Xu H, He Z, Feng K-Y, Hu L, Cui W, Wang K, Dong X, Xie L, Kong X, Cai Y-D, Li Y: **Prediction of Deleterious Non-Synonymous SNPs Based on Protein Interaction Network and Hybrid Properties**. *PLoS ONE* 2010, **5**:e11900.
