# Supplementary material for: Differential combinatorial regulatory network analysis related to venous metastasis of hepatocellular carcinoma
Source: BMC Genomics. 2012 Dec 17;13(Suppl 8):S14. doi: 10.1186/1471-2164-13-S8-S14 (PMC3535701; doi:10.1186/1471-2164-13-S8-S14)
Supplement: Additional file 2 — Performance comparison between module-based and gene-list-based classifiers. [file 1471-2164-13-S8-S14-S2.pdf]

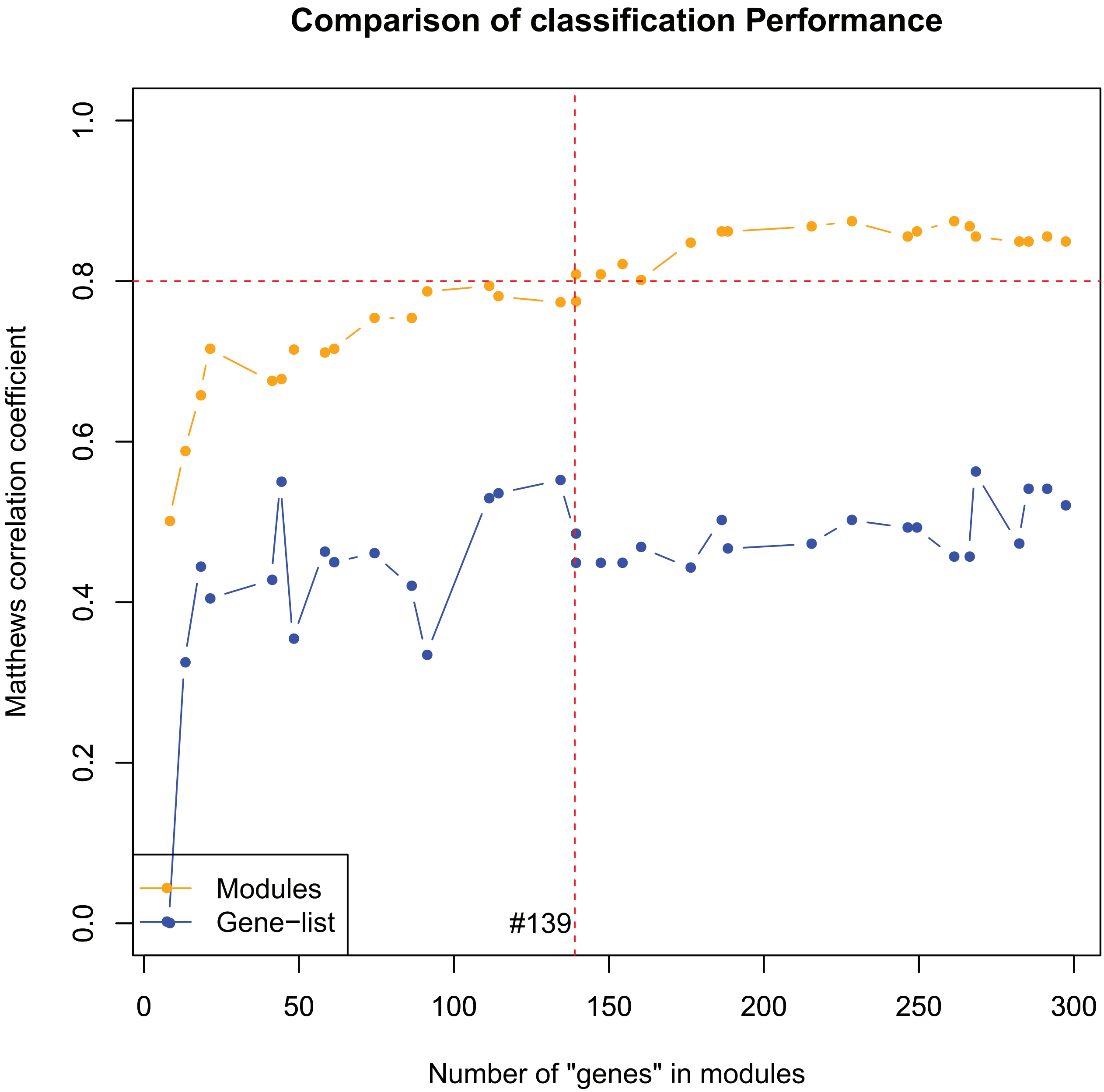

We repeated the classification procedure by adding one more candidate module at a time from the previously prepared ranked list of candidate modules until the total number of genes reached 300. Also, each time we included the same number of genes picked from the top of the MRMR-ranked gene list into the gene-list-classifier. The x axis represents the number of "genes" involved in the modules, including TFs, miRNAs, or genes, and the y axis represents the value of Matthew correlation coefficient(MCC). One dot on a line represents addition of one more module. When the number of modules accumulated to 17, ACC overrides 90% and MCC overrides 80%.
